# Quantitative Proteomics Identifies Metabolic Pathways Affected by *Babesia* Infection and Blood Feeding in the Sialoproteome of the Vector *Rhipicephalus bursa*

**DOI:** 10.3390/vaccines8010091

**Published:** 2020-02-19

**Authors:** Joana Couto, Margarita Villar, Lourdes Mateos-Hernández, Joana Ferrolho, Gustavo S. Sanches, Ana Sofia Santos, Maria Margarida Santos-Silva, João Nobre, Olga Moreira, Sandra Antunes, José de la Fuente, Ana Domingos

**Affiliations:** 1 Instituto de Higiene e Medicina Tropical, Universidade Nova de Lisboa, Rua da Junqueira, 100, 1349-008 Lisboa, Portugal; joana.ferrolho@ihmt.unl.pt (J.F.); GustavoSeron@hotmail.com (G.S.S.); santunes@ihmt.unl.pt (S.A.); adomingos@ihmt.unl.pt (A.D.); 2 Global Health and Tropical Medicine, Instituto de Higiene e Medicina Tropical, Universidade Nova de Lisboa (GHTM-IHMT-UNL), Rua da Junqueira, 100, 1349-008 Lisboa, Portugal; 3 SaBio, Instituto de Investigación en Recursos Cinegéticos IREC-CSIC-UCLM-JCCM, Ronda de Toledo s/n, 13005 Ciudad Real, Spain; MargaritaM.Villar@uclm.es (M.V.); lmateoshernandez@hotmail.com (L.M.-H.); jose_delafuente@yahoo.com (J.d.l.F.); 4 Biochemistry Section, Faculty of Science and Chemical Technologies, and Regional Centre for Biomedical Research (CRIB), University of Castilla-La Mancha, 13071 Ciudad Real, Spain; 5 UMR BIPAR, ANSES, INRA, Ecole Nationale Vétérinaire d’Alfort, Université Paris-Est, F-94700 Maisons-Alfort, France; 6 Instituto Nacional de Saúde Doutor Ricardo Jorge, Centro de Estudos de Vectores e Doenças Infecciosas Dr. Francisco Cambournac (CEVDI), Av. ª da Liberdade, 5, 2965-575 Águas de Moura, Portugal; ana.santos@insa.min-saude.pt (A.S.S.); m.santos.silva@insa.min-saude.pt (M.M.S.-S.); 7 Instituto de Saúde Ambiental, Faculdade de Medicina, Universidade de Lisboa, Av. Prof. Egas Moniz, 1649-028 Lisboa, Portugal; 8 Instituto Nacional de Investigação Agrária e Veterinária, Quinta da Fonte Boa, Vale de Santarém, 2005-048 Santarém, Portugal; joao.nobre@iniav.pt (J.N.); olga.moreira@iniav.pt (O.M.); 9 Department of Veterinary Pathobiology, Center for Veterinary Health Sciences, Oklahoma State University, Stillwater, OK 74078, USA

**Keywords:** ticks, *Babesia*, proteomic, RNAi, UB2N, PCCA

## Abstract

The negative impact of ticks and tick-borne diseases on animals and human health is driving research to discover novel targets affecting both vectors and pathogens. The salivary glands are involved in feeding and pathogen transmission, thus are considered as a compelling target to focus research. In this study, proteomics approach was used to characterize *Rhipicephalus* *bursa* sialoproteome in response to *Babesia* *ovis* infection and blood feeding. Two potential tick protective antigens were identified and its influence in tick biological parameters and pathogen infection was evaluated. Results demonstrate that the *R. bursa* sialoproteome is highly affected by feeding but infection is well tolerated by tick cells. The combination of both stimuli shifts the previous scenario and a more evident pathogen manipulation can be suggested. Knockdown of *ub2n* led to a significative increase of infection in tick salivary glands but a brusque decrease in the progeny, revealing its importance in the cellular response to pathogen infection, which is worth pursuing in future studies. Additionally, an impact in the recovery rate of adults (62%), the egg production efficiency (45.75%), and the hatching rate (88.57 %) was detected. Building knowledge on vector and/or pathogen interplay bridges the identification of protective antigens and the development of novel control strategies.

## 1. Introduction

Ticks have a significant negative impact on host species through their feeding behavior, causing direct skin and sub-cutaneous tissue damage and blood depletion, and also acting as vectors of different pathogens such as viruses, bacteria, and protozoa [1,2]. Belonging to the Ixodidae family, *Rhipicephalus bursa* is a multi-host tick widely distributed in the Mediterranean region having cattle, sheep, and goats as its primary hosts but can occasionally be found in wild ungulates, small mammals, or even humans [3,4,5]. This tick species is the main vector of the etiological agent of ovine babesiosis, *Babesia ovis*. This tick-borne disease affects small ruminants and is prevalent in Eastern Asia, Southern Europe (Mediterranean basin), Middle East, and Northern Africa, overlapping *R. bursa* geographical distribution [6,7]. Ovine babesiosis is an acute disease whose onset is characterized by high fever, that can progress to other clinical symptoms such as hemolytic anemia, hemoglobinuria, icterus, and in severe cases, pancytopenia. Untreated cases usually lead to death and even upon treatment the animal may die as the result of a heavy infection or suffer disease relapse after the withdrawal of therapy [8,9,10]. Despite an established enzootic situation in countries such as Iran, fatal disease outbreaks have been reported in Spain and particularly in Turkey, demonstrating the deleterious effect of *B. ovis* in naïve sheep transferred from a tick-free region to a *R. bursa*-infested region with endemic babesiosis [11,12,13]. As in other babesiosis, disease control relies on chemotherapy with imidocarb dipropionate to manage clinical symptoms, and on vector control using acaricides [14,15]. Both these strategies have major drawbacks in the host, such as safety issues concerning animal-derived food products as milk contamination but also the potential carcinogenicity of imidocarb [16,17]. Furthermore, acaricide resistance and its detrimental impact in the environment [14,15,18] supports the need for safer alternatives for disease control. A deeper understanding of tick biology and tick-pathogen interactions is fundamental to identify candidate protective antigens that can be targeted to reduce vector competence and ultimately control babesiosis.

Pathogens have co-evolved and adapted to survive within the tick vector cells by regulating host processes such as the acquisition of nutrients, modification of the host environment, and meddling with immune responses [18,19,20,21,22,23] that could be targeted for the identification of protective antigens [24,25]. After entering the vector, pathogens need to disseminate through tick tissues, infect and multiply within salivary gland (SG) cells to be successfully transmitted to susceptible hosts during tick blood meal [23]. Tick SGs are morphologically complex organs with multifunctional roles in different biological processes such as osmoregulation, feeding, and pathogen transmission [1,26,27,28,29,30,31,32,33,34]. Tick salivary compounds, or sialome, include a plethora of molecules essential to counteract host immune reaction to tick attachment and feeding, including anti-platelet aggregator compounds, anticoagulants, and vasodilators that will be released to host bloodstream via saliva [1,34]. SGs are also responsible for the production of cement cone related proteins which are not only accountable for an efficient attaching but also show antimicrobial properties and act against the host immune system [35,36].

Previous studies focused on tick sialome, aiming to characterize the transcriptome and proteome for different tick species and recently the sialotranscriptome response of adult *R. bursa* to *B. ovis* infection has been investigated [37]. As in other tick species, results confirmed the complexity of the SG transcriptomics response to different conditions such as pathogen infection and feeding [37,38] leading to the synthesis of a wide range of proteins [26,37].

Thus, proteomics approach was used in the present study to obtain first, information regarding the SG protein composition and second, to evaluate the sialoproteome in response to blood feeding and pathogen infection. The present study constitutes the first *R. bursa* sialoproteome report, demonstrating the dynamic changes occurring in the tick-pathogen interface. Understanding the SG molecular dynamics is a key for the discovery of pharmacologically active compounds of clinical interest such as protective antigens for anti-tick and pathogen transmission blocking vaccines.

## 2. Materials and Methods

### 2.1. Ethics Statement

This study was carried out with the approval of the Divisão Geral de Alimentação e Veterinária (DGAV), Portugal, (under Art° 49, Portaria n°1005/92 from 23rd October, permit number 0421/2013) and the Council of Ethics of the Instituto de Higiene e Medicina Tropical (IHMT). Animals experiments were conducted in accordance with the national and European Animal Welfare legislation (in frame with DL 113/2013 and Directive 2010/63/EU) and the principle of the Three R’s, to replace, reduce, and refine the use for scientific purposes.

### 2.2. Rhipicephalus Bursa Tick Colony

According to the described protocol [39], established *R. bursa* colony was fed in white rabbits (strain Hyla) and for moulting kept in a chamber regulated at 25 ± 1 °C, 70 ± 10% relative humidity, and a photoperiod of 16:8 (light:dark) at Instituto Nacional de Saúde Doutor Ricardo Jorge. After oviposition and during the two generations, eggs and ticks were tested for pathogens (*Babesia* spp., *Anaplasma* spp., *Ehrlichia* spp.) by PCR, using the protocols and primers described elsewhere [40,41,42,43]. Pathogen-free progeny was then used to establish the tick colony.

### 2.3. Babesia Ovis Culture

*Babesia ovis* (Israeli strain) were maintained in vitro at the Institute of Hygiene and Tropical Medicine (IHMT) as previously described by Antunes et al. 2018 [37].

### 2.4. Infection and Feeding of Rhipicephalus Bursa Ticks

**Figure 1:**
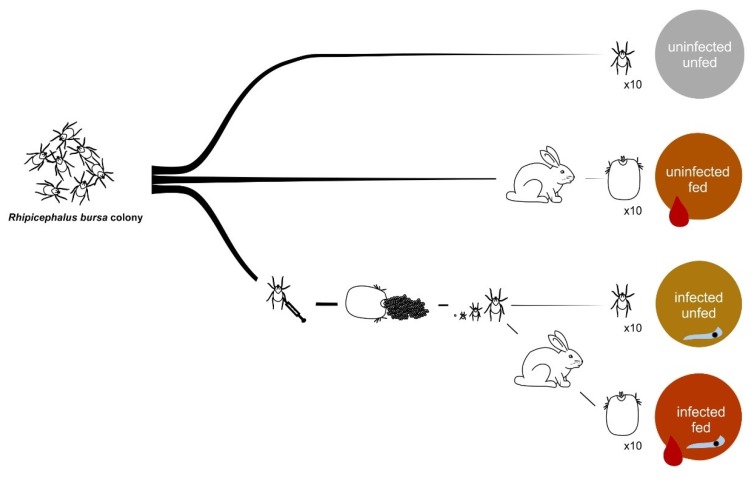
Experimental design for production of ticks at different feeding and infection conditions. From a pathogen-free colony, four groups of 10 *R. bursa* females were generated in triplicate: uninfected unfed (grey circle), uninfected fed (orange circle), infected unfed (yellow circle), and infected fed (red circle). The uninfected unfed ticks were obtained directly from the colony; and the uninfected fed ticks were carefully detached from the rabbit ear after blood meal. Infection of groups were achieved by inoculation of *B. ovis* in female ticks. After egg laying and hatching, infected batch of larvae developed to the adult phase. To obtain the infected unfed group, ticks were directly used, and to obtain infected fed groups, ticks were allowed to feed. Finally, tick salivary glands were dissected to perform the proteomics analysis of the *R. bursa* sialoproteome.

The experimental design concerning the production of female *R. bursa* ticks is described in Figure 1. Briefly, four groups of ticks were generated: uninfected unfed (NINF), uninfected fed (NIF), infected unfed (INF), and infected fed (IF). Uninfected unfed ticks from the colony were used to obtain NINF group and adult female ticks feeding on rabbits were carefully removed from the rabbit ear 6-8 days post attachment to produce the NIF group. To produce *B. ovis* infected ticks, female adult ticks were directly inoculated in the first leg articulation of trochanter-coxae with *B. ovis* from a 15–20% infected blood culture and allowed to feed in rabbits. After drop-off, females were kept under the rearing conditions described above. Progenies were tested for *B. ovis* as described elsewhere [44]. Infected larvae were allowed to feed in order to obtain adults. A part of infected batch of female ticks were fed to obtain the IF group and the remaining ticks were used to produce the INF group.

### 2.5. Tick Dissection, DNA Extraction, and B. ovis Infection

First, ticks were rinsed individually in distilled water and 75% (*v*/*v*) ethanol. Salivary glands (SG) of *R. bursa* females were dissected in ice-cold phosphate-buffered saline (PBS) under a stereomicroscope and stored in RNA later (Ambion, Austin, TX, USA) at −20 °C. DNA was extracted using TRI-Reagent^®^ (Sigma–Aldrich, MO, USA). SG infection was evaluated using the above referred protocol [44].

### 2.6. Protein Extraction and Trypsin Digestion

SG were homogenized with a 20 gauge needle in lysis buffer (7 M Urea, 2 M Thiourea, 2% 3-[(3-cholamidopropyl) dimethylammonio]-1-propanesulfonate, CHAPS). Samples were sonicated for 1 min in an ultrasonic cooled bath and vortexed for 10 s. After three cycles of sonication-vortex, the homogenates were centrifuged at 200× *g* for 5 min to remove cellular debris. The supernatants were collected, and protein concentration was determined using the RC-DC protein assay (Bio-Rad, CA, USA) with BSA as standard.

Protein extracts (200 µg) were precipitated and digested as performed by Artigas-Jerónimo and colleagues [45], until the peptides were finally desalted onto OMIX Pipette tips C18 (Agilent Technologies, CA, USA), dried-down and stored at −20 °C until downstream applications.

### 2.7. Proteome Analysis by SWATH-MS

The desalted protein digests were resuspended in 2% acetonitrile with 5% acetic acid and analyzed by reverse phase liquid chromatography coupled online with mass spectrometry (RP-LC-MS/MS) using an Ekspert nLC 415 system combined to a 6600 TripleTOF^®^ mass spectrometer (AB SCIEX^®^, MA, USA) through information-dependent acquisition (IDA) followed by sequential windowed data independent acquisition of the total high-resolution mass spectra (SWATH). Approximately 5 µg of each protein digest from each replicate sample were pooled together as a mixed sample from each group (uninfected unfed, uninfected fed, infected unfed, and infected fed). Pooled mixed samples were then used for the generation of the reference spectral ion library as part of SWATH-MS analysis. For details, see Supplementary Material and Methods S1.

### 2.8. Library Generation/Protein Identification, Data Processing and Relative Quantitation

A spectral library of all the detectable peptides in the samples and relative quantitation was performed according to Estrada-Peña [46], with the exception of spectra identification which was performed by searching against a compiled database containing all sequences from Ixodidae and *Babesia* taxonomies and rabbit (*Oryctolagus cuniculus*) proteome (135,071, 19,087 and 21,178 Uniprot entries, respectively, in September, 2017) with the following parameters: iodoacetamide cysteine alkylation, trypsin digestion, identification focus on biological modification, and thorough ID as search effort. The MS raw proteomics data have been deposited at the PeptideAtlas repository (http://www.peptideatlas.org/) with the dataset identifier PASS01362. All the identified proteins and quantitation data are represented in the Supplementary spreadsheet.

### 2.9. Gene Ontology

Gene ontology was obtained using Blast2GO software (version 3.0.11, available at http://www.blast2go.org) [47,48]. Homology to the protein identification (UniprotID) was searched by blast against Arthropoda (nr subset) [arthropoda, taxa:6656] from 30.01.2017 as well as a mapping and annotation steps to assign functional terms at level 3. GO terms were also assigned manually based on UniProt-associated databases. GO frequency and protein regulation charts were constructed for each condition using the Microsoft Office 2016 Excel tool. To elucidate about the GO and the differentially representation of proteins in response to infection, feeding, or both, chord diagrams were generated using the GOplot R package in RStudio (Version 1.1.453) [49].

### 2.10. In Silico Analysis of Proteins, Selection of Targets and Recombinant Protein, and Peptide Production

Proteins commonly represented in the four conditions were further characterized using the software STRING 10.5 (Search Tool for the Retrieval of Interacting Genes/Proteins available at http://string-db.org) in order to identify known/novel protein–protein interactions in the *Ixodes scapularis* database. Briefly, the program generates the network images based on a spring model. The selection of targets for further analysis was based on three main criteria: (1) Proteins present in all the datasets from proteomics; (2) proteins that may have a pivotal role in tick-parasite interplay; and (3) proteins that may be potential protective antigens resulting in a vaccine candidate. The amino acid sequences of L7M1X7 and L7MAU7 were analyzed in silico in order to predict the protein localization (CELLO v.2.5 [50]), transmembrane domains (TMHMM v.2.0, based on a hidden Markov model [51,52]), signal peptides (SignalP v.5.0 [53]), antigenic determinants (using the method developed by Kolaskar and Tongaonkar [54] available at http://imed.med.ucm.es/Tools/antigenic.pl), solubility (PROSOII [55]), and crystallizability (SECRET [56]). With a purity higher than 75%, a recombinant protein (based on L7M1X7, UB2N, 151 a.a.) and a peptide (VKTPEECVKIAQSIGYPVMIKASAGGGGKGMRIAWND based on L7MAU7, PCCA, 37 a.a.) were selected to be synthetically produced by GenScript Corporation (Piscataway, NJ, USA) for the polyclonal antibodies production and immunoassays. To increase peptide immunogenicity, an Imject™ Blue Carrier™ Protein (highly soluble, mollusc-derived hemocyanin) (Pierce Biotechnology Inc., IL, USA) was conjugated with the peptide using one step glutaraldehyde conjugation [57]. Protein concentration was assessed by spectrophotometry using a NanoDrop ND-1000 (Thermo Scientific, MA, USA) and samples were analyzed by SDS-PAGE. Briefly, 10 µg of peptide or Imject™ Blue Carrier™ Protein or recombinant protein and 50 μg of protein extracts from each condition were re-suspended in Laemmli buffer (Bio-Rad, CA, USA) containing 5% (*v*/*v*) of 2-β-mercaptoethanol, separated on a 12.5% or 4–20% discontinuous SDS-PAGE gels.

### 2.11. Hybridoma and Polyclonal Antibody Production

Polyclonal antibodies were obtained by immunization of 3–4 weeks-old CD1 male mice (reared in IHMT) with the recombinant protein or conjugated peptide. For each target, three CD1 male mice were primed and boosted intraperitoneally every 2–3 weeks with 20 µg of protein or conjugated peptide emulsified with incomplete Freund’s adjuvant (Sigma–Aldrich, MO, USA) in a 1:1 proportion. Pre-immune serum was collected prior to immunization and serum from mandibular vein blood was collected before each inoculation to monitor anti-target antibodies titers by indirect ELISA. The mouse with higher antibody titer was selected and 3 days before euthanasia a final boost was given. Spleens from the selected animals were collected, as well as total blood, to further obtain spleen cells and antiserum, respectively. Spleen cells were used to fuse with Sp2/0 myeloma cells (ATCC) (previously cultured in DMEM media, supplemented with 10% of fetal bovine serum (Gibco, Invitrogen Corporation, Paisley, UK)) at a ratio of 1:1 in the presence of polyethylene glycol (PEG, Sigma–Aldrich, MO, USA). Hybridoma cells were selected in DMEM media, 10% (*v*/*v*) fetal calf serum (Biowest, MO, USA) supplemented with hypoxanthine-aminopterin-thymidine HAT (Sigma–Aldrich, MO, USA) and subsequently cloned by limiting dilution technique. Cell cultures were maintained at 37 °C in a 5 % CO_2_ incubator. Clones producing the highest titers of specific antibodies, as assessed in the indirect ELISA and Western blot, were selected for further use.

### 2.12. Indirect ELISA and Western Blot

To determinate the antibody titer of mice serum and cell supernatant, the ELISA protocol described by Couto et al. (2017) [58] was employed with minor modifications: a high binding 96-well ELISA plate (Corning^®^ Costar^®^, MA, USA) was coated with 0.1 µg of peptide or protein diluted in PBS. Mice serum (diluted 1:200 in PBS) or cell supernatant (without dilution) was incubated for one hour at 37 °C. Antibody capacity to recognize specific targets was assessed by Western blot. After SDS-PAGE, the proteins were transferred overnight at a constant 25 V to a nitrocellulose membrane, with a pore size of 0.2 μm (Bio-Rad, CA, USA), using the Mini Trans-Blot^®^ Electrophoretic Transfer Cell (Bio-Rad, CA, USA). Membranes were stained with Ponceau S (Figure S5) and polyacrylamide gels with BlueSafe (NZYTech, Lisbon, PT) to validate the transfer process. Later, membranes were blocked with 5% (*w*/*v*) non-fat dry milk (Bio-Rad, CA, USA) in PBS containing 0.05% (*v*/*v*) Tween 20 (PBS-T) at room temperature (RT) for one hour. After washing with Tris-buffered saline complemented with 0.05% (*v*/*v*) Tween 20 (TBST), membranes were incubated with mouse serum (1:200) or a hybridoma supernatant (without dilution), for 2 h at RT. After three 15-min washes with TBST, membranes were incubated for 1 h with alkaline phosphatase-conjugated anti-mouse polyvalent immunoglobulins (G, A, M) (1:3000; Sigma–Aldrich, MO, USA) or a goat anti-mouse IgG (H+L)-horseradish peroxidase conjugated secondary antibody (1:2000; Bio-Rad, CA, USA). The antigen–antibody complexes were detected using the alkaline phosphatase (AP) conjugate substrate kit (Bio-Rad, CA, USA) or ECL Western blotting detection reagent (ECL, GE Healthcare Life Sciences, PA, USA) exposed for 10 s and 10 min on a Hyperfilm (GE Healthcare Life Sciences, PA, USA). Validation of differential protein representation on SG protein extracts used for proteomic analysis was performed by Western blot using polyclonal serum and hybridomas produced. Protein band intensities were estimated using ImageJ Software (version 1.51K). Additionally, the *pcca* and *ub2n* expression was also assessed using qPCR in infected and naïve SGs of fed *R. bursa* ticks, following the protocol described below.

### 2.13. Synthesis of dsRNA and RNAi Assays

RNA obtained from *R. bursa* females was used to synthesize cDNA using the iScript™ cDNA Synthesis Kit (Bio-Rad, CA, USA), and the cDNA was subsequently used to amplify a region of interest of each mRNA sequence from unrelated mouse beta-2-microglobulin (*β2m*), *pcca* and *ub2n* genes using specific primers containing T7 promoter sequences at the 5′- end (Supplementary Table S2) [59,60] according to previous studies. Females ticks were injected with 138 nL of dsRNA (with 1.38 × 10^11^ molecules) following the protocol described in other studies [37,61].

Gene silencing assays were performed to evaluate the effect of the genes that code for L7M1X7 and L7MAU7 proteins on tick biological parameters and *Babesia* infection. The conditions of this assays were previously described in Antunes et al. 2018 [37]. Briefly, a splenectomised, six-month-old lamb was maintained and fed *ad libitum* at the Instituto Nacional de Investigação Agrária e Veterinária animal facility. The lamb was intravenously inoculated with 3 mL of cryopreserved *B. ovis* culture with 6% of parasitemia and infection was monitored using qPCR described in the following section. In parallel, a *R. bursa* colony reared and maintained in IHMT was used to obtain adult ticks. Four groups were generated: two control groups (unrelated mouse beta-2-microglobulin dsRNA, dsβ2M, and non-inoculated), a group targeting UB2N and another targeting PCCA. Each group included fifty female ticks that were previously injected with gene-specific dsRNA and fifty male ticks to allow mating to further analyze reproductive-related parameters. Infestation was performed during infection peak and ticks were allowed to feed for 9 days in a specific tick-feeding cell. Ticks were monitored daily and dropped ticks were collected. After 9 days, attached ticks were manually removed. Ticks were randomly selected for two purposes: ten ticks were dissected to further evaluate gene knockdown efficiency and infection rate in the SG, and the remaining ticks were maintained under controlled conditions to evaluate the biological parameters of the progeny and transovarial transmission of *Babesia*.

### 2.14. Gene Expression and Knockdown Assessment

To evaluate gene expression and knockdown efficiency through qPCR, ten female ticks were randomly selected per group and its SG dissected as previously described. Total RNA and DNA were extracted from each sample using TRI-Reagent^®^. RNA quantity was determined using the ND-1000 Spectrophotometer (NanoDrop ND1000) and its integrity was evaluated using the Qubit™ RNA IQ Assay Kit in the Qubit™ 4 Fluorometer (Thermo Scientific, MA, USA). RNA concentrations of 1 µg/µL were used for cDNA synthesis with iScript™ cDNA Synthesis Kit (Bio-Rad, CA, USA) in a T100 Thermal Cycler (Bio-Rad, CA, USA). Using a CFX Connect™ Real-Time PCR Detection System (Bio-Rad, CA, USA), *ub2n* and *pcca* expression was assessed. The following conditions were used: initial cycle of denaturation at 95 °C for 10 min; followed by 45 cycles of 95 °C for 15 s and temperature of each primer set for 30 s (Supplementary Table S3) [59,60]; and finally a dissociation curve (55–95 °C, 0.5 °C/s). Negative controls and standard curves (constructed with ten-fold serial dilutions) were included in each qPCR to validate reaction specificity and determine the PCR efficiency. The average expression stability (M-value) of the reference genes (Supplementary Table S3) [62] and gene relative quantification were assessed based in the geNorm algorithm [63] and the Pfaff method [64], respectively, included in the CFX Manager™ Software (Bio-Rad, CA, USA).

### 2.15. Babesia Ovis Quantification

In order to evaluate the infection in the host blood as well as in tick SG after feeding and in progeny, absolute quantification of *B. ovis* was assessed by qPCR. Genomic DNA was extracted from 200 µL of blood collected at day 32, 34, 35, 37, 38, 41, 44, and 46 from the lamb; and from ticks SG and larvae, using TRI-Reagent^®^ as described above. qPCR reactions of 10 μL were performed in triplicate using SYBR Green Supermix kit (Bio-Rad, CA, USA) in a CFX Connect™ Real-Time PCR Detection System (Bio-Rad). The following conditions were used: Initial cycle of denaturation at 95 °C for 5 min; followed by 49 cycles of 95 °C for 10 s and temperature of each primer set for 30 s (Supplementary Table S4) [59,60]; and finally a dissociation curve (55–95 °C, 0.5 °C/s). Reaction specificity was validated by including negative controls using RNase-free water as template. To determine the PCR efficiency and gene copy number, synthetized gBlocks^®^ Gene Fragments (Integrated DNA Technologies, Leuven, BE) (Supplementary Table S4) [65] were used to produce standard curves with ten-fold serial dilutions. Based on Dandasena et al. (2018) [66], copy number was calculated using the Equation (1):

(1) $$number\;of\;copies\;\left(molecules\right)=\frac{a\;ng\times 6.0221x10^{23}}{\;b\;g/mol\times 1x10^{9\;}ng/g}\;\left(1\right)$$

a: mean of quantity obtain from qPCR, b: target molar mass.

*Babesia* infection in the host was evaluated as the ratio of copy number of *BoSPD*/*Ov18S* genes, whereas in the vector it was evaluated by *BoSPD*/*16S* genes. *Babesia* infection was compared between dsRNA-inoculated groups using the non-parametric Mann-Whitney test (SPSS v24.0) [67]. Logarithm (10 based) was applied to evaluated the percentage of increase or reduction of infection.

### 2.16. Tick Biological Parameters

Recovery rate (RR, as the percentage of the ratio between live ticks and the total number of female ticks), drop-off (DO, as the percentage of the ratio between ticks that dropped-off and the total number of female ticks), and engorged female weight (EFW) were determined in order to elucidate about tick fitness, while reproductive parameters such as egg mass weight (EMW), egg production efficiency (EPE, as percentage of the ratio between EMW and EFW), and egg hatching rate (EHR, as the mean value of visual evaluation performed by five technicians separately) were analyzed after dropped-off females laid the eggs. The data are expressed as mean ± standard deviation, and statistical significance was determined using SPSS v24.0 [67] (*p < 0.05, **p < 0.01, ***p < 0.001). Chi-square and Phi and Cramer’s V tests were used to evaluate the level of association of RR and DO between dsRNA-inoculated groups. Normality and homogeneity of variance were first checked using Shapiro-Wilk’s and Levene’s tests, respectively. Mann-Whitney and t-test were used as non-parametric and parametric tests.

## 3. Results and Discussion

### 3.1. R. Bursa Sialoproteome

The main objective of this study was the identification of candidate tick protective antigens based on the characterization of the *R. bursa* sialoproteome in response to blood feeding and *B. ovis* infection. Four conditions with 10 *R. bursa* females each were produced in triplicate, to understand the processes of infection when ticks are unfed or fed, as well as the effect of feeding when ticks are uninfected and infected (Figure 1).

**Figure 2:**
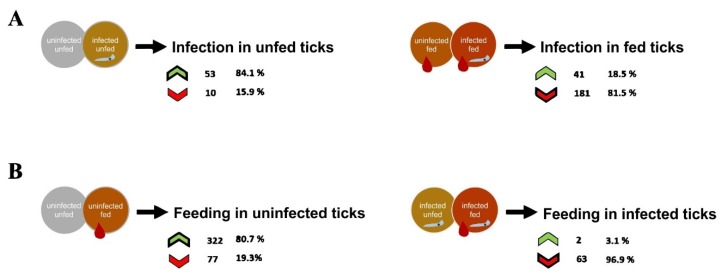
Differentially represented proteins from the *R. bursa* sialoproteome after infection (**A**) and blood feeding (**B**). For each condition it is represented the process of infection (parasite) and feeding (blood drop), as well as the number and percentage of proteins differentially over (green arrow) and under (red arrow) represented in *R. bursa* salivary glands. Grey circle + no symbols = uninfected and unfed ticks, yellow circle + parasite = infected and unfed ticks, orange circle + blood drop = uninfected and fed ticks, red circle + parasite + blood drop = infected and fed ticks.

SG were dissected from all groups for DNA and protein extraction, to perform respectively, *B. ovis* detection and proteomics analysis. The PCR detected *Babesia* DNA in all samples from infected groups, confirming the infection of these ticks (Supplementary Figure S1). Proteomics analysis of tick SGs were carried out resulting in the identification of a total of 1617 proteins, in which a high percentage of proteins (98.08 %) corresponded to the tick vector. After excluding the host and parasite-related proteins, from the 1586 tick proteins identified, 585 differentially represented proteins were found (*p* < 0.05) in response to blood feeding or parasite infection and used for further characterization (Figure 2).

The results showed that while infection in unfed ticks resulted in a higher number of over-represented than under-represented proteins, in fed ticks the number of under-represented proteins increased and was higher than the number of over-represented proteins (Figure 2A). In uninfected ticks, blood feeding resulted in a higher number of over-represented proteins but in infected ticks blood feeding reduced the levels of a larger number of proteins (Figure 2B). These results suggested that the response to *Babesia* infection and blood feeding leads to an increase in tick vector protein levels when acting independently, but the combination of both stimuli overcomes this effect by reducing protein levels in response to feeding and infection.

**Figure 3:**
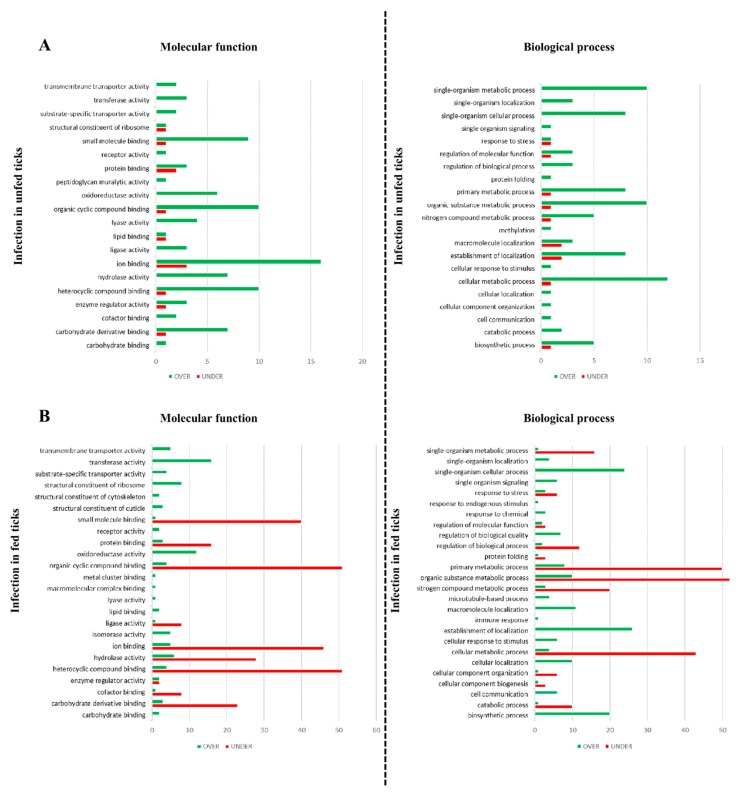
Gene ontology of the effect of *B. ovis* infection in unfed (**A**) and fed (**B**) *R. bursa* ticks. Functional terms at level 3 were assigned based on UniProt and associated databases. Green bars = over-represented proteins, red bars = under-represented proteins.

**Figure 4:**
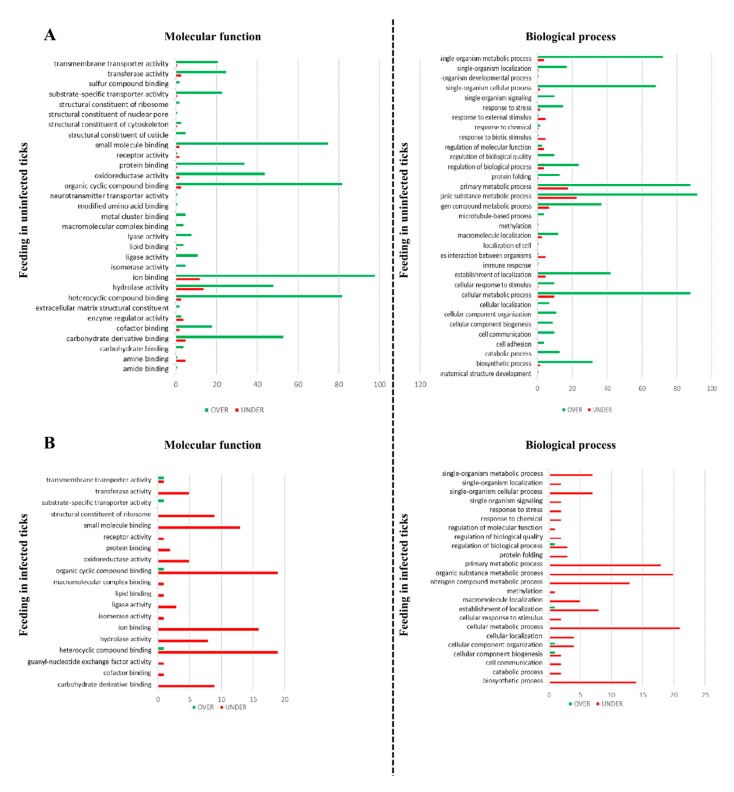
Gene ontology of the effect of feeding in uninfected (**A**) and *B. ovis* infected (**B**) *R. bursa* ticks. Functional terms at level 3 were assigned based on UniProt and associated databases. Green bars = over-represented proteins, red bars = under-represented proteins.

Gene ontology (GO) annotation was assessed for each UniProt ID obtained from the *R. bursa* proteome using Blat2GO and UniProt-related databases. Of the 1586 identified-proteins, only 97 proteins were classified as “unknown” due to the absence of GO and domain function. The remaining 1489 annotated proteins were classified according to the GO terms molecular function (MF), biological process (BP), and cellular component (CC) at level 3. Focusing on the 585 differentially represented proteins, the representation of GO terms in each condition is shown in Figure 3 and Figure 4 and Supplementary Figures S2 and S3.

### 3.2. Effect of Babesia Infection on R. bursa Sialoproteome

To characterize the effect of *B. ovis* infection on *R. bursa* SG, proteomics data from uninfected unfed ticks was compared to the infected unfed group (Figure 2A and Figure 3A and Supplementary Figure S2A).

In Figure 2A, a set of 63 proteins differentially represented were obtained, where 84.1% and 15.9% were significantly over- and under-represented proteins, respectively. Such lower number of differently represented proteins suggest that *Babesia* might influence *R. bursa* SGs translation but at a lower rate because of its parasitic relationship [18]. Focusing on the GO, the sialoproteome during infection presented more proteins linked to cell and membrane part, membrane-bounded organelle (CC) (Supplementary Figure S2A), heterocyclic and organic cyclic compound binding, ion binding, small molecule binding (MF) (Figure 3A), cellular, organic substance, and single-organism metabolic processes but also with establishment of localization (BP) (Figure 3A). Additionally, some GO terms were exclusively constituted by over-represented proteins and none exclusively under-represented (Figure 3A). Peptidoglycan muralytic activity was a GO term exclusive of this process of infection in unfed ticks, being constituted by an over-represented protein (UniProt ID: L7M9R2), presenting domains that are found among peptidoglycan recognition proteins being associated to innate immunity and conserved between insects and mammals [68]. By recognizing microbial particles and activating antimicrobial defense systems such as prophenoloxidase and Toll receptor cascade, it is possible to induce the production of antimicrobial peptides [68] that have a negative effect on parasite multiplication and survival [69]. Together with the presence of only a GO term (GO term: “response to stress”) associated to cellular response (N = 2), as well as the absence of proteins related to “regulation of biological quality” and “response to chemical,” this cellular response suggest that *Babesia* is recognized by *R. bursa* SG cells in a moderate manner, producing lower levels of such proteins because of its evolutionary relationship [70,71].

### 3.3. Effect of Blood Feeding on R. bursa Sialoproteome

A perspective of feeding process was obtained with the comparison of SG proteome from uninfected unfed and uninfected fed groups (Figure 2B and Figure 4A and Supplementary Figure S3A).

In this set, from the 399 differently represented proteins, 80.7% and 19.3% were found to be over and under-represented, respectively (Figure 2B), belonging to a wide range of functional classes (Figure 4A and Supplementary Figure S3A). In our previous work concerning *R. bursa* sialotranscriptome, the cellular machinery was highly activated when uninfected ticks undergo blood feeding demonstrated that during *R. bursa* feeding SG shifts from an inactive to a metabolically active status with intense gene transcription prevailing gene over expression (75% up regulated) [37]. Overall, both studies clearly demonstrate that blood ingestion requires a high production of cellular molecules, which is reflected by the high expression of genes and its subsequent translation. In this set of proteins, multiple GO terms are exclusively over-represented. Moreover several proteins were exclusively found in this dataset, being associated to amide and amine binding, extracellular matrix structural constituent, modified amino acid binding, neurotransmitter transporter activity, structural constituent of nuclear pore, and sulphur compound binding (MF), as well as anatomical structural development, cell adhesion, interspecies interaction between organisms, localization of cells, response to biotic and external stimulus, and finally, single-organism development process (BP). Besides that, the GO terms related to “structural constituent of cuticle,” “cell-cell junction,” and “anatomical structural development” confirms the investment of blood feeding in tick engorgement and development [72].

### 3.4. Effect of Infection in the Sialoproteome of Fed R. bursa

To evaluate the process of infection in fed ticks, the infected fed and uninfected fed sialoproteomes were compared (Figure 2A and Figure 3B and Supplementary Figure S2B).

A high number of proteins (N = 222) were found significantly represented, with 81.5% being under-represented (Figure 2A). Tick feeding is a process that demands the synthesis of a high number of molecules [37,73] in which transcription and translation appears to be correlated resulting in an over expression and over representation of both transcripts and proteins. In contrast, in the presence of parasite infection this does not seems to occur. While, sialotranscripts of fed *R. bursa* during *B. ovis* infection that were analyzed in Antunes study [37] demonstrated that its majority is up regulated (64%), in the present study the majority of the differentially represented proteins are under-represented suggesting that infection in fed ticks may influence translation and ultimately protein production. Exposure to infection in fed ticks resulted in the production of proteins linked to diverse GO terms (Figure 3B and Supplementary Figure S2B). The most represented GO terms of CC and MF categories are the same when ticks are fed or unfed, being more under-represented when tick have a blood meal. At BP level, cellular, organic substance and single-organism metabolic processes are the most represented GO terms (Figure 3B), constituted by more proteins under-represented. Some GO terms are entirely over-represented, being only six CC GO terms associated exclusively to under-represented proteins (protein, ribonucleoprotein and transporter complex, supramolecular polymer, virion part and whole membrane). “Response to endogenous stimulus” was a GO term only present in this condition of infection in fed ticks. Besides, other two GO term related to stress response were identified (“response to stress” and “response to chemical”). This suggests that the production of proteins that mediate cellular response to stimuli is being stimulated only when blood meal occurs.

### 3.5. Effect of Feeding in the Sialoproteome of B. ovis Infected R. bursa

By comparing the infected fed and infected unfed sialoproteomes, a feeding process is analyzed when ticks are subjected to infection (Figure 2B and Figure 4B and Supplementary Figure S3B).

In this context, from a total of 65 proteins, 96.9% proteins were under-represented (Figure 2B). The most represented GO terms are represented in Figure 4A and Supplementary Figure S3A, with more proteins under-represented than in the uninfected dataset. The majority of GO terms includes exclusive under-represented proteins, except DNA packing and protein-DNA complex, outer and whole membrane (CC), and finally substrate-specific transporter activity (MF) that are exclusively over-represented. Interestingly, when ticks are exposed to both *Babesia* infection and blood meal (infection in fed ticks and feeding in infected ticks), over-represented proteins with DNA packaging and protein-DNA complex properties are commonly presented. Such proteins influence the transcription and translation mechanism resulting in a decrease in the production of proteins as shown before.

### 3.6. R. bursa Cellular Machinery in Response to Feeding and Infection

**Figure 5:**
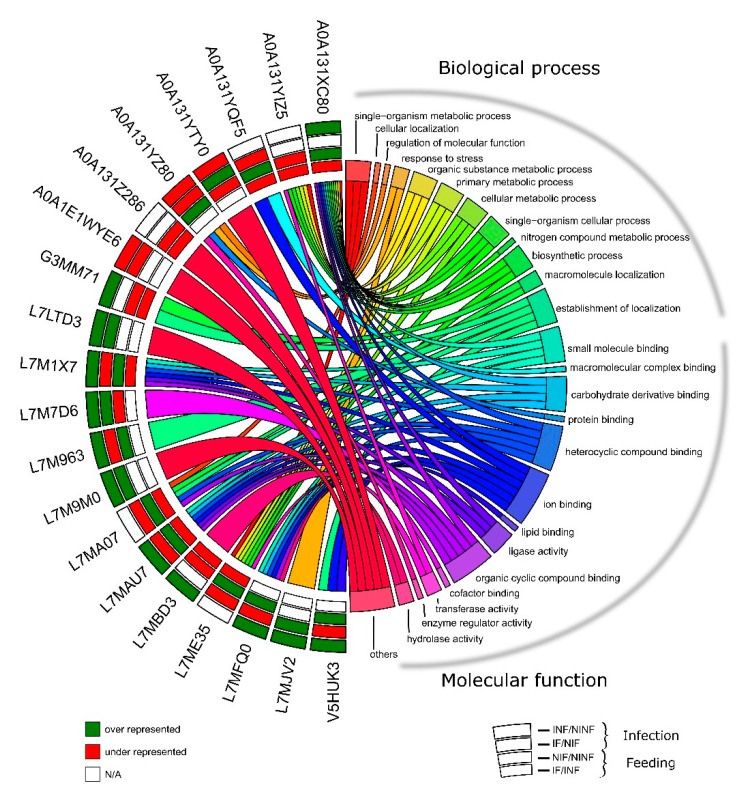
Chord diagram presenting gene ontology of the differentially represented proteins in *R. bursa* sialoproteome in response to infection and/or feeding. Each protein found in each comparison is shown on the left alongside with UniProt ID, while the GO clusters are shown on the right. Outer annulus to inner annulus: infection in unfed ticks (INF/NINF), infection in fed ticks (IF/NIF), feeding in uninfected ticks (NIF/NINF), and feeding in infected ticks (IF/INF). Green square: over-represented, red square: under-represented and white square: not applicable.

To build knowledge about the response of R. bursa SG, the datasets were analyzed to identify proteins commonly represented at the different conditions (Supplementary Table S1). Figure 5 summarizes the proteins involved in infection, feeding, or both.

The process of infection (when ticks are fed or unfed) modulate the representation of twelve proteins of *R. bursa* SGs, while blood meal (when ticks are infected or uninfected) influence ten proteins. From these, two proteins, a putative ubiquitin-protein ligase (UniProt ID: L7M1X7, UB2N) and an uncharacterized protein (UniProtID: L7MAU7, PCCA), were found in all comparisons, its representation being modulated positively when ticks are exposed to a single stimulus (INF/NINF and NIF/NINF) and negatively when ticks are exposed to both stimuli (IF/NIF and IF/INF) (Figure 5, Supplementary Table S1). According to the defined criteria for target selection for RNA interference studies, in silico analysis were performed revealing characteristics such as the putative function, subcellular localization, and immunogenicity of these proteins. STRING analysis showed that those targets and their network are linked to ubiquitination and metabolic pathways (Supplementary Figure S4), essential for tick fitness [21,74] and described as drug targets in other contexts [75,76]. CELLO, TMHMM, and SignalP servers predicted the cytoplasmatic localization and absence of transmembrane helices or signal peptides in both proteins, that alongside with their antigenic propensity (UB2N: 1.0357, with 8 antigenic determinants; PCCA: 1.0357, with 28 antigenic determinants) indicate that those proteins could be tested to evaluate their potential as protective antigens.

### 3.7. The Role of a Putative Ubiquitin-Protein Ligase in R. bursa and B. ovis Interface

**Figure 6:**
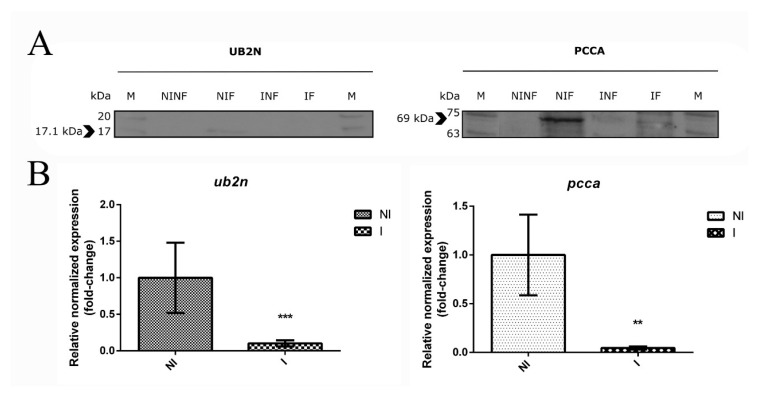
Protein representation and gene expression of selected targets. (**A**) Western blot of UB2N and PCCA. Protein extracts from salivary glands exposed to different conditions were used to validate the protein representation of UB2N and PCCA by using mouse serum (1:200) and a hybridoma supernatant (without dilution), respectively. Arrows indicate the molecular size of the target. M: molecular weight, NZYColour Protein Marker II, NZYTech. NINF: uninfected unfed. NIF: uninfected fed. INF: infected unfed. IF: infected fed. Exposure and contrast parameters were not modified. The full-length blots are displayed in Supplementary Figure S5. (**B**) Relative expressions of *ub2n* and *pcca* were evaluated in fed uninfected and fed *B. ovis* infected salivary glands of *R. bursa* using qPCR. Data was normalized using 16S rRNA, *elongation factor*, and *β-tubulin* reference genes. The expression of fed uninfected group (control) is set to 1 for a better interpretation. NI: fed uninfected group. I: fed infected group. Statistical analysis were conducted using the Pfaff method. Significance is represented by ** *p* < 0.01, *** *p* < 0.001.

Ubiquitination is a biological process that affects proteins by adding to them ubiquitin moieties [77]. This process could influence proteins by altering their cellular location, activity, and interaction with other molecules, being involved in pleiotropic roles such as protein degradation [78], cell–cell communication [79], pathogen invasion [80], and innate immune system [80]. Ubiquitin addition involves the sequential action of three main groups of enzymes: ubiquitin-activating (E1s), ubiquitin-conjugating (E2s), and ubiquitin-ligase (E3s) enzymes [79]. An E1 enzyme interacts with an E2 that subsequently coupled with a specific E3 leading to ubiquitin incorporation in a protein sequence. The putative ubiquitin-protein ligase, UB2N (UniProt ID: L7M1X7) possesses several domains that are common among E2s (CDD: cd00195, InterPro: IPR023313 and IPR000608) reflecting its function as an ubiquitin-conjugating enzyme. This 17.1 kDa protein was found and validated as positively regulated in response to feeding or infection stimulus alone (Proteomics: INF/NINF = 0.57, NIF/NINF = 0.49; Western blot: NIF/NINF = 78347.143/ND), while occurrence of both processes, feeding and infection, lead to its negative regulation (Proteomics: IF/NIF = −1.07, IF/INF = −1.15; Western blot: IF/NIF = ND/78347.143) (Supplementary Table S1, Figure 6A).

**Figure 7:**
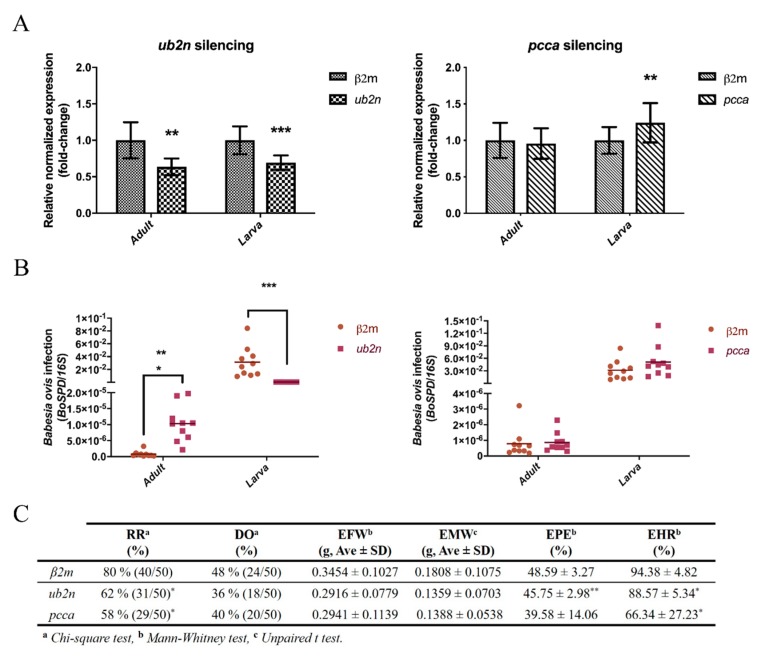
Effect of dsRNA-inoculation in *R. bursa* during *B. ovis* infection. (**A**) Gene knockdown assessment by measuring *ub2n* and *pcca* relative expressions in dsRNA-inoculated ticks. Data were normalized using 16S rRNA, *elongation factor*, and *β-tubulin* reference genes. The expression of *β2m*-inoculated group (control) is set to 1 for a better interpretation. Statistical analysis were conducted using the Pfaff method. (**B**) *B. ovis* infection in salivary glands (Adult) and progeny (Larvae) of dsRNA-inoculated female *R. bursa* ticks. A ratio between copy number of *BoSPD* and 16SrRNA for each sample in each condition is represented. Statistical analysis were conducted using the Mann-Whitney test. (**C**) Evaluation of tick biological parameters after dsRNA inoculation. Data are represented as percentage, ratio, means, and standard deviation. Statistical analysis were conducted using the Chi-squared, Mann-Whitney, and Student’s *t* tests. RR: recovery rate, DO: drop-off, EFW: engorged female weight, EMW: egg mass weight, EPE: egg production efficiency, EHR: egg hatching rate. Significance is represented by * *p* < 0.05, ** *p* < 0.01, *** *p* < 0.001.

Also, qPCR results demonstrates that the *ub2n* transcript was down regulated (qPCR: 0.126, *p* value < 0.001, Figure 6B) during infection when ticks are fed, suggesting no impact of the translation process [81] in protein levels. Such directional regulation of UB2N and pivotal role in the cell machinery and pathogen colonization reflects its potential as a protective vaccine candidate, as described in other studies for other ubiquitination-related proteins [74]. This regulation could be a way of SG cells to overcome a specific event such as feeding or infection by stimulating the ubiquitination pathway in order to achieve homeostasis and cellular protection [79,80]. However, when dealing with various extracellular threats, cells become sensitive to several stimuli [82] and could be influenced by manipulative organisms [80] such as *Babesia*. Considering this, RNAi assays were conducted in order to evaluate the impact of *ub2n* knockdown in *B. ovis* infection (in SGs and progeny) and tick fitness (Figure 7).

Regarding the adult ticks, ds*ub2n* inoculation resulted in a significant reduction of *ub2n* mRNA levels (0.636; *p* = 0.010) in SG with a silencing efficiency of 36.4% (Figure 7A). Moreover, *ub2n* silencing lead to a significant increase in *B. ovis* infection in *R. bursa* SGs (18.32 %, *p* < 0.001) (Figure 7B), suggesting the UB2N as a protective molecule against *Babesia*. Shaw and colleagues also demonstrated that the reduction of ubiquitin-related enzymes gene expression hampered protection against *Anaplasma phagocytophilum* infection leading to an increase of bacteria load [74]. Regarding tick biological parameters (Figure 7C), results show that *ub2n* silencing did not influence female engorgement (EFW, *p* value = 0.104) and drop-off (DO, X2 = 1.478, *p* value = 0.224). This absence of impact in tick feeding was previously demonstrated after silencing an ubiquitin-ligase enzyme (XIAP) [74]. The mortality rate increased significantly (RR, *p* value = 0.047, Phi and Cramer’s V: weak/moderate) suggesting an essential role of UB2N in tick survival. Additionally, results show that *ub2n* silencing did not influence significantly egg weight (EMW, *p* value = 0.102) (Figure 7C). However, a negative influence in egg production (EPE, *p* value = 0.001) and its viability (EHR, *p* value = 0.045) was observed in ds*ub2n*-inoculated group. Assessment of gene knockdown in the progeny of dsRNA inoculated ticks showed a significant reduction of *ub2n* expression (0.693; *p* < 0.001) in larvae with a silencing efficiency of 30.7% (Figure 7A) confirming that gene silencing can be perpetuated through future generations. While *Babesia* infection increased in SGs, the opposite occurred in larvae suggesting an impact in *Babesia* transovarial transmission since the parasite load decreased abruptly (−138.53%, *p* < 0.001) (Figure 7B). The hypothesis of *ub2n* expression stabilization that could stimulate the IMD signalling pathway promoting antimicrobial peptides production [83] capable to control apicomplexan infection [69] is discarded in this context since the silencing efficiency was similar to the adult phase. Such decrease of infection could be explained by a putative effect of *ub2n* silencing in ovaries, i.e., could have blocked the invasion of *B. ovis* in the ovaries and consequently through the progeny.

The results of the present study indicate that even with a silencing efficiency of about 30%, ticks and parasite were significantly affected in both stages. Overall, UB2N demonstrated to be a key molecule in tick biology with an important role on the cellular response to pathogen infection worth to pursue in future studies, specially evaluate its effect in ovary development.

### 3.8. New Insights about an Uncharacterized Protein

Biotin-dependent carboxylases, which includes a major group that uses as substrate coenzyme A (CoA), e.g., acetyl-CoA carboxylase (ACCA), propionyl-CoA carboxylase (PCCA), and 3-methylcrotonyl-CoA carboxylase (MCCA) [84], are key molecules in several metabolic pathways influenced during tick-pathogen interplay including fatty acid, amino acid, and carbohydrate metabolisms [21,22]. Such enzymes are considered attractive targets for drug discovery against several diseases, including bacterial and fungal infections [85,86]. The previously mentioned uncharacterized protein (UniProtID: L7MAU7, PCCA) has similarities to the sequence and domains of a propionyl-CoA carboxylase alpha chain protein from *Rhipicephalus appendiculatus* (E-value: 0.0, Identity: 96.1%), thus its function as a PCCA enzyme can be assumed. In most organisms, this carboxylase catalyzes the conversion of a glucose precursor propionyl-CoA to D-methylmalonyl-CoA in the mitochondrial matrix, playing a role in the catabolism of β-branched amino acids, cholesterol side chain and fatty acids [84]. During feeding, catabolism of molecules allows the use of smaller elements in anabolic reactions required for tick development. In our proteomics analysis, this 69.5 kDa protein was found over-represented in response to feeding stimulus alone (Proteomics: NIF/NINF = 0.30; Western blot: NIF/NINF = 99915.597/ND) and also in response to infection (Proteomics: INF/NINF = 0.38; Western blot: INF/NINF = 9234.421/ND) (Supplementary Table S1, Figure 6A). This positive regulation suggests a role of PCCA enzyme as a key for energy supply, maybe through the formation of building blocks or nutrients that ultimately contributes to tick and parasite growth [21]. Moreover, previous studies reported that bacteria and fungi metabolize and detoxify propionyl-CoA by the 2-methylcitrate cycle [87] to overcome its toxicity and growth inhibition properties [88]. In ticks such detoxification and cell growth maintenance could be achieved by PCCA since this carboxylase catalyzes the propionyl-CoA. Interestingly, when feeding and infection processes are combined, the protein levels of PCCA in tick SGs (Proteomics: IF/NIF = −0.48, IF/INF = −0.55; Western blot: IF/NIF = 22503.948/99915.597) (Supplementary Table S1, Figure 6A) as well as the gene expression decrease (qPCR: 0.058, *p* value < 0.001, Figure 6B). We hypothesize that such reduction of PCCA culminate in a toxic environment to tick cells as well as growth inhibition, facilitating *Babesia* dissemination since this apicomplexan parasite could use an alternative way such as the 2-methylcitrate cycle to surpass toxic environments and cell growth inhibition in order to pursue infection dissemination. Based on this and considering the potential of carboxylases as versatile targets for drug discovery against apicomplexan infections [85,89,90,91], silencing assays were conducted in order to evaluate the influence of *pcca* mRNA reduction on *Babesia* infection and tick biological parameters (Figure 7). In the conditions undertaken in the present study, *pcca* gene knockdown was not achieved (0.957; *p* = 0.285) (Figure 7A) with only 4.3 % reduction of mRNA levels. Also, the progeny of *pcca* dsRNA inoculated ticks revealed that *pcca* expression increased significantly (1.242; *p* = 0.010). Further studies are required to clarify if PCCA has a role in the metabolic pathways related to tick development that could be influenced by infection.

## 4. Conclusions

The numerous proteins detected in the *R. bursa* SG highlight the complexity of the processes in this issue. The dynamic response of *R. bursa* SG to feeding, infection, and to both stimuli was characterized, pinpointing the potential tick antigens involved in relevant tick biological functions. RNAi assays place UB2N as an important protein in the cellular response to pathogen infection in *R. bursa,* which should be further explored. The putative role of PCCA in the evaluated tick parameters and infection is not disclosed herein; however future experiments using different conditions should be performed to characterize it.

## Supplementary Materials

The following are available online at https://www.mdpi.com/2076-393X/8/1/91/s1. Figure S1, Agarose gel electrophoresis for validation of *Babesia* infection in *Rhipicephalus bursa* salivary glands. Figure S2, Gene ontology based on cellular component of the effect of *Babesia* infection in unfed (**A**) and fed (**B**) *Rhipicephalus bursa* ticks. Figure S3, Gene ontology based on cellular component of the effect of feeding in uninfected (**A**) and infected (**B**) *Rhipicephalus bursa* ticks. Table S1, Representation of the fold-change of proteins found in the *Rhipicephalus bursa* sialome at different experimental conditions. Figure S4, Predicted networks of UB2N and PCCA proteins. Table S2, Primers used for double-stranded RNA synthesis. Table S3, Primers used for gene knockdown assessment. Table S4, Primers used for detection and quantification of *Babesia ovis*. Figure S5, Protein representation of selected targets analyzed by Western blot.
