# Supplementary material for: Quantitative Proteomics Identifies Metabolic Pathways Affected by Babesia Infection and Blood Feeding in the Sialoproteome of the Vector Rhipicephalus bursa
Source: Vaccines (Basel). 2020 Feb 19;8(1):91. doi: 10.3390/vaccines8010091 (PMC7157752; doi:10.3390/vaccines8010091)
Supplement: Supplementary file 1 [file vaccines-08-00091-s001.pdf]

vaccines

# Supplementary Material and Methods S1. Extended detailroteome analysis by SWATH-MS

The peptides were concentrated using a 0.1 × 20 mm C18 RP pre-column (Thermo Scientific, MA, USA), and then separated using a 0.075 × 250 mm C18 RP column (New Objetive, MA, USA) operating at 0.3 µl/min. Peptides were eluted using a 120-min gradient from 10 to 30 % solvent B followed by 10-min gradient from 30 to 40 % solvent B (Solvent A: 0,1 % formic acid in water, solvent B: 0,1 % formic acid in acetonitrile) and directly injected into the mass spectrometer for analysis. For IDA experiments, the mass spectrometer was set to scan full spectra (390-1400 m/z) using 250 ms accumulation time per spectrum, followed by up to 50 MS/MS scans (100-1500 m/z). Candidate ions with a charge state between +2 and +5, and counts per second above a minimum threshold of 100, were isolated for fragmentation. One MS/MS spectra was collected for 100 ms, before adding those precursor ions to the exclusion list for 15 s (mass spectrometer operated by Analyst® TF 1.6, AB SCIEX®). Dynamic background subtraction was turned off. MS/MS analysis were recorded in high sensitivity mode with rolling collision energy on and a collision energy spread of 5. For SWATH quantitative analysis, 10 µg of each independent sample were subjected to the cyclic Data Independent Acquisition (DIA) of mass spectra using the SWATH variable windows calculator (V 1.0, AB SCIEX®) and the SWATH acquisition method editor (AB SCIEX®), following previously established methods [46]. A set of 50 overlapping windows was constructed (containing 1 m/z for the window overlap), covering the precursor mass range of 400–1250 m/z. For these experiments, a 50 ms survey scan (390–1400 m/z) was acquired at the beginning of each cycle, and SWATH-MS/MS spectra were collected from 100–1500 m/z for 70 ms at high sensitivity mode, resulting in a cycle time of 3.6 s. Collision energy for each window was determined according to the calculation for a charge +2 ion-centered upon the window with a collision energy spread of 15.

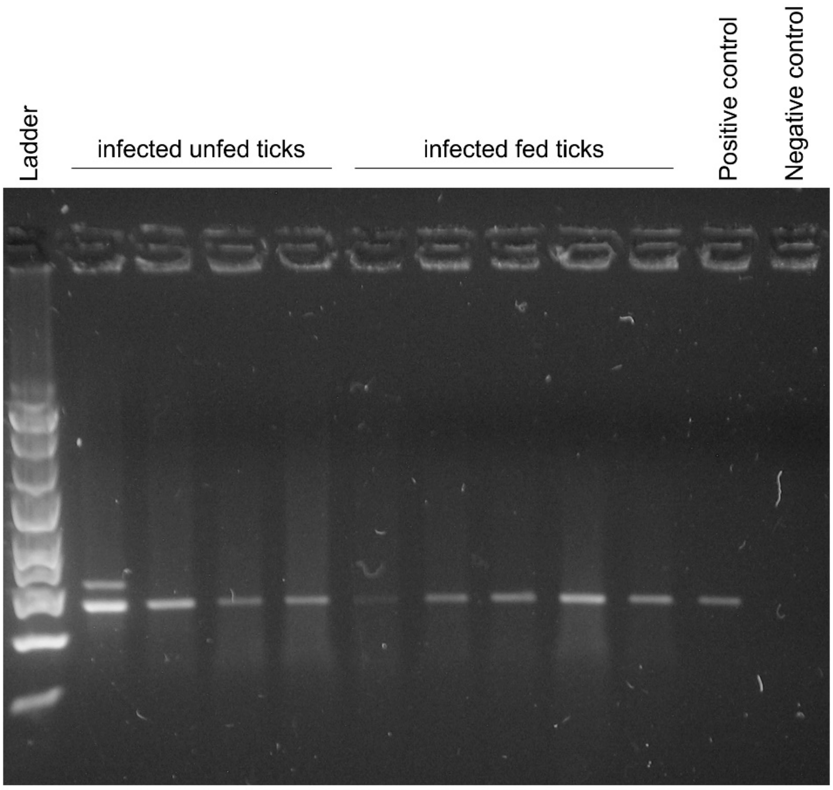


**Figure S1. Agarose gel electrophoresis for validation of *Babesia* infection in *Rhipicephalus bursa* salivary glands.** Lane 1: NZYDNA Ladder VIII ranging from 200 to 5000 bp; Lanes 2-5: PCR product

derived from DNA extracted from individual infected unfed ticks; Lanes: 6-10: PCR product derived from DNA extracted from individual infected fed ticks; Lanes 11: positive control; Lane 12: negative control. Bands with approximately 600 bp were considered as positive for the amplification of 18S rRNA of *Babe*sia in this full-length gel.

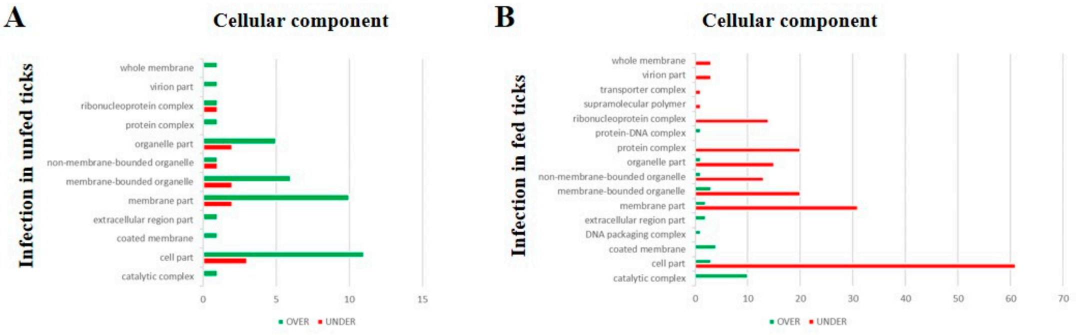


**Figure S2.** Gene ontology based on cellular component of the effect of *Babesia* infection in unfed (A) and fed (B) *Rhipicephalus bursa* ticks. Green bars = over represented proteins, red bars = under represented proteins.

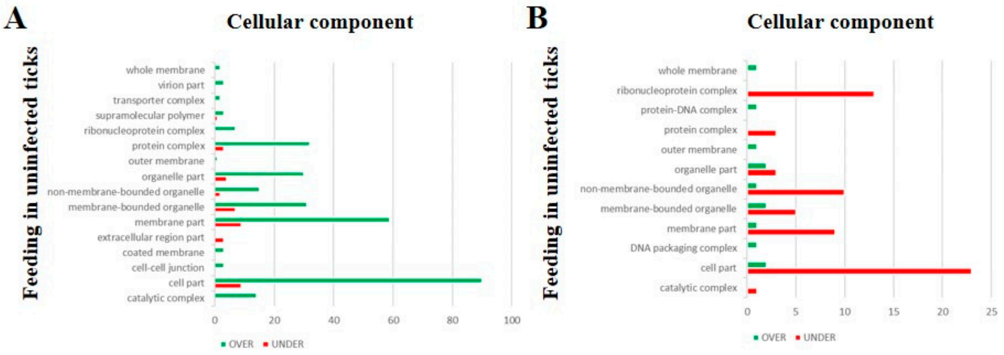


**Figure 3.** Gene ontology based on cellular component of the effect of feeding in uninfected (A) and infected (B) *Rhipicephalus bursa* ticks. Green bars = over represented proteins, red bars = under represented proteins.

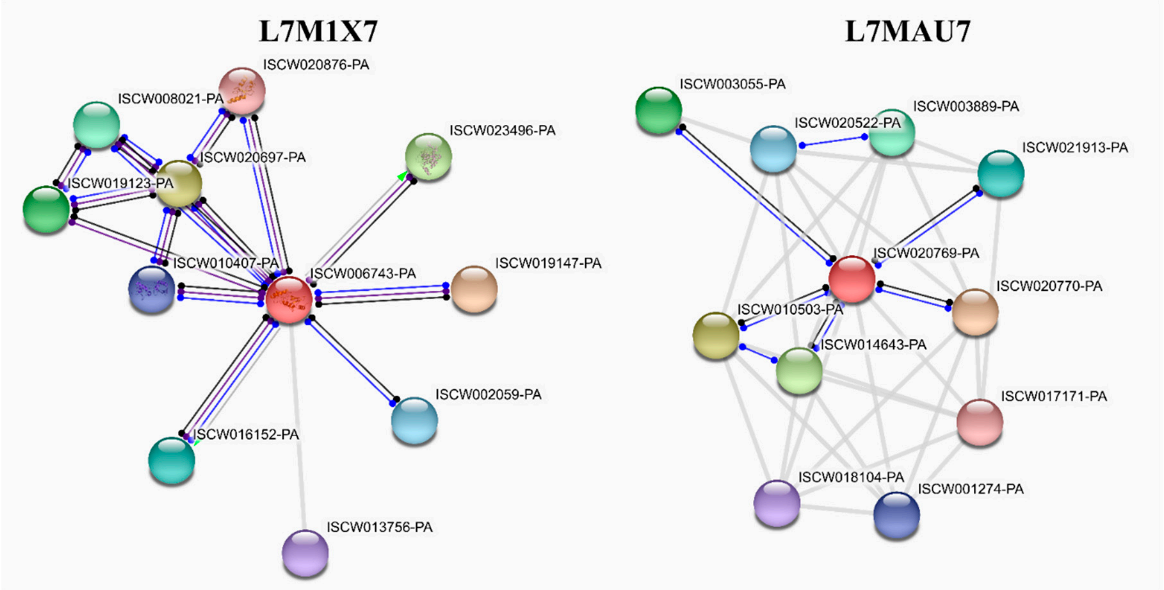


**Figure S4. Predicted networks of UB2N and PCCA proteins.** STRING-based network prediction analysis considering a minimum required interaction score of 0.900 (highest confidence). Unspecified action types are predicted such as binding (blue), reaction (black) and catalysis (purple). Based on KEGG, Pfam and InterPro databases, UB2N (UniProt ID: L7M1X7) is linked to an ubiquitin mediated proteolysis, while PCCA (UniProt ID: L7MAU7) to several metabolic pathways (Propanoate metabolism, Valine, leucine and isoleucine degradation, Carbon metabolism, Glyoxylate and dicarboxylate metabolism, Metabolic pathways and Microbial metabolism in diverse environments).

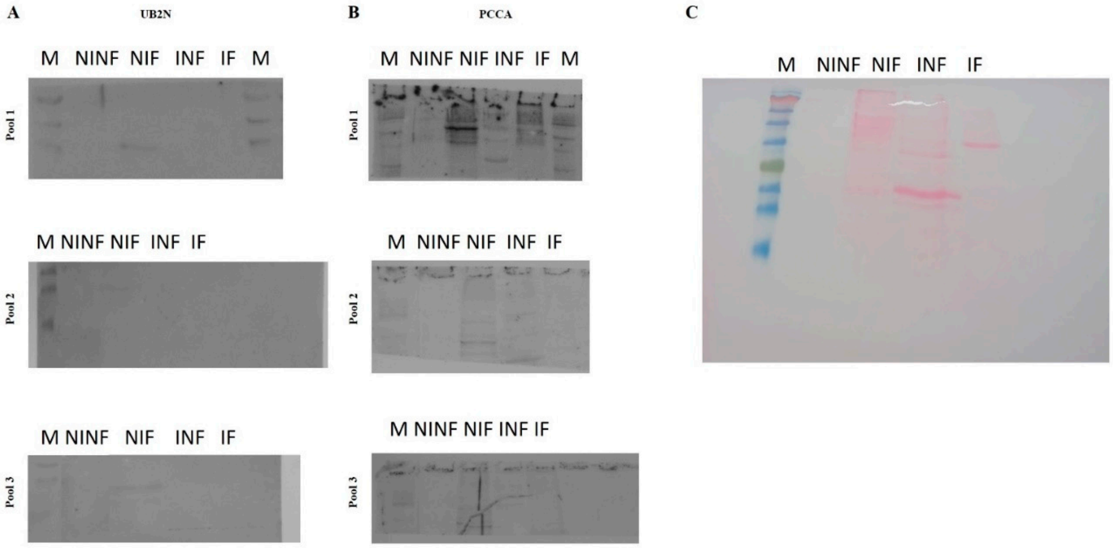


**Figure S5. Protein representation of selected targets was analysed by western blot. A)** Western blot of UB2N (N=3) and **B)** of PCCA (N=3). Protein extracts from salivary glands exposed to different conditions were used to validate the protein representation of UB2N and PCCA by using mouse serum (1:200) and a hybridoma supernatant (without dilution), respectively. **C)** Representative Ponceau staining of a nitrocellulose membrane. M: molecular weight, NZYColour Protein Marker II, NZYTech. NINF: uninfected unfed. NIF: uninfected fed. INF: infected unfed. IF: infected fed. Exposure and contrast parameters were not modified. The cropped blots regarding the specific molecular size of each target of pool 1 are displayed in Figure 6.

**Table S1.** Representation of the fold-change of proteins found in the *Rhipicephalus bursa* sialome at different experimental conditions. For each protein, its description, UniprotID code and fold-change (log base 10) is provided at different conditions.

| Description | UniProt ID | Infection in unfed ticks | Infection in fed ticks | Feeding in uninfected ticks | Feeding in infected ticks |
|---|---|---|---|---|---|
| **Putative tpa exp: histone 1 (Fragment)** | A0A023FRV8 | | 0.58 | | 0.25 |
| **Putative acetyl-coa acetyltransferase** | A0A023FW52 | | -1.52 | 0.54 | |
| **Signal transducing adaptor molecule** | A0A0B4PMI2 | | -0.55 | 0.39 | |
| **Putative cop9 signalosome subunit csn8** | A0A0C9S0W8 | | -0.55 | 0.73 | |
| **Putative electron transfer flavoprotein alpha subunit (Fragment)** | A0A0C9SC62 | | -0.48 | 0.31 | |
| **Putative threonyl-trna synthetase (Fragment)** | A0A131XC80 | 0.45 | | 0.36 | -0.33 |
| **Putative grp-3 498 glycine rich family (Fragment)** | A0A131XFS3 | 0.41 | | | -0.77 |
| **Putative mitochondrial ribosomal protein l12 strongylocentrotus purpuratus** | A0A131XJN3 | 0.62 | | | -0.55 |
| **Putative electron transfer flavoprotein beta subunit** | A0A131XL69 | | -0.49 | 0.48 | |
| **Putative flotillin** | A0A131XLN9 | 0.41 | | 0.37 | |
| **Signal recognition particle 54 kDa protein (Fragment)** | A0A131XLX5 | | -1.04 | | -0.68 |
| **Putative ubiquinone oxidoreductase ndufb6/b17 subunit (Fragment)** | A0A131XMC0 | | -0.36 | 0.32 | |
| **Putative ksr 2 misexpression suppressor** | A0A131XMP5 | | -0.59 | 0.41 | |
| **Putative lipocal-1 1** | A0A131XNF5 | | 1.18 | -1.40 | |
| **Determination of adult lifespan** | A0A131Y981 | | 0.53 | -0.92 | |
| **Uncharacterized protein** | A0A131YFB0 | | -0.60 | 0.33 | |
| **Glutathione S-transferase** | A0A131YGX4 | | -0.66 | | -0.70 |
| **F-type H+-transporting ATPase subunit f** | A0A131YHA2 | 0.47 | | 0.34 | |
| **Uncharacterized protein** | A0A131YI07 | | -0.40 | | -0.57 |
| **Translocating chain-associated membrane protein 1** | A0A131YI36 | 0.44 | | | -0.58 |
| **RNA-binding protein Musashi** | A0A131YIH8 | | -0.56 | 0.57 | |
| **Chitin-binding protein** | A0A131YIS0 | | 0.96 | -1.16 | |
| **Dolichyl-diphosphooligosaccharide--protein glycosyltransferase subunit 1 (EC 2.4.99.18)** | A0A131YIZ5 | | | -0.31 | -0.20 |
| **Glucosylceramidase (EC 3.2.1.45)** | A0A131YJ49 | | 0.75 | -0.87 | |

| Description | UniProt ID | Infection in unfed ticks | Infection in fed ticks | Feeding in uninfected ticks | Feeding in infected ticks |
|---|---|---|---|---|---|
| **Reprolysin** | A0A131YJK3 | | -0.86 | 1.26 | |
| **Acetyl-CoA acyltransferase 2** | A0A131YJK6 | | -0.52 | 0.54 | |
| **Pancreatic trypsin inhibitor** | A0A131YKR1 | | 1.00 | -1.05 | |
| **Carbonic anhydrase** | A0A131YKX5 | 0.43 | | 1.06 | |
| **Ubiquitin carboxyl-terminal hydrolase 7** | A0A131YLU4 | | 0.07 | | -0.34 |
| **Lethal(2) giant larvae protein** | A0A131YM12 | | -0.24 | 0.17 | |
| **TIL domain containing protein** | A0A131YMT5 | | 0.60 | -0.64 | |
| **Large subunit ribosomal protein L41** | A0A131YP01 | | -0.64 | 0.75 | |
| **Reductase** | A0A131YP17 | 0.71 | | | -0.86 |
| **EF hand domain-containing protein** | A0A131YPL5 | | 0.76 | -1.06 | |
| **Isocitrate dehydrogenase (NAD+)** | A0A131YPL8 | | -0.43 | 0.38 | |
| **LETM1 and EF-hand domain-containing protein 1, mitochondrial** | A0A131YQF5 | | -1.24 | 0.27 | -0.98 |
| **Basic tail secreted protein** | A0A131YQH0 | 0.78 | | 1.18 | |
| **Chaperonin GroEL** | A0A131YRI2 | | -0.42 | 0.47 | |
| **Proteasome subunit beta type (EC 3.4.25.1)** | A0A131YRZ3 | | -0.46 | 0.66 | |
| **Complement component 1 Q subcomponent-binding protein, mitochondrial** | A0A131YSG0 | | -0.40 | 0.33 | |
| **Rhipicephalus family xi** | A0A131YTY0 | -0.18 | 0.93 | -1.37 | |
| **Proteasome subunit beta type (EC 3.4.25.1)** | A0A131YV56 | | -0.56 | 0.70 | |
| **Poly [ADP-ribose] polymerase (PARP) (EC 2.4.2.30)** | A0A131YW69 | | -0.54 | 0.20 | |
| **TIL domain containing protein** | A0A131YWN5 | | 0.50 | | -0.47 |
| **Elongation factor Tu** | A0A131YXL2 | | -0.43 | 0.36 | |
| **Ankyrin** | A0A131YXX3 | | -0.63 | 0.36 | |
| **Activator of 90 kDa heat shock protein atpase log 1** | A0A131YZ80 | -0.76 | -1.09 | 0.51 | |
| **Aconitate hydratase, mitochondrial (Aconitase) (EC 4.2.1.-)** | A0A131Z0E6 | | -0.51 | 0.55 | |
| **Arginine-rich protein** | A0A131Z0L8 | | -0.39 | 0.45 | |
| **Eukaryotic translation initiation factor 3 subunit I (eIF3i)** | A0A131Z255 | | -0.16 | | -0.16 |
| **Chitin-binding protein** | A0A131Z267 | | 0.20 | | -0.24 |
| **Glycine rich secreted protein** | A0A131Z286 | | | -0.99 | -0.68 |
| **Proteasome subunit beta type (EC 3.4.25.1)** | A0A131Z2F3 | | -0.67 | 0.48 | |

| Description | UniProt ID | Infection in unfed ticks | Infection in fed ticks | Feeding in uninfected ticks | Feeding in infected ticks |
|---|---|---|---|---|---|
| **ATP-binding cassette, subfamily C (CFTR/MRP), member 5** | A0A131Z2I8 | 0.26 | | | -0.87 |
| **Antigen 5 family member** | A0A131Z2P4 | | 1.43 | -1.59 | |
| **Coatomer subunit gamma** | A0A131Z2X8 | | -0.73 | 0.54 | |
| **Phosducin like 3** | A0A131Z3X0 | | -0.74 | 0.65 | |
| **Reticulon-like protein** | A0A131Z4F1 | | -0.75 | 0.49 | |
| **TIL domain containing protein** | A0A131Z4H4 | 0.43 | | 1.56 | |
| **Sodium/potassium-transporting ATPase subunit beta** | A0A131Z5K7 | | -0.43 | 0.58 | |
| **Cystatin-A/B** | A0A131Z6C6 | | -1.02 | 0.45 | |
| **Proteasome subunit beta (EC 3.4.25.1)** | A0A131Z8C6 | | -0.38 | 0.35 | |
| **Small subunit ribosomal protein S28e (Fragment)** | A0A131ZB20 | | -0.43 | | -0.16 |
| **Protein disulfide isomerase family A, member 5 (Fragment)** | A0A131ZC65 | | -0.78 | 0.59 | |
| **Putative aconitase/aconitase aconitase superfamily protein (Fragment)** | A0A147BVC5 | | -0.80 | 0.58 | |
| **Uncharacterized protein** | A0A1E1WYE6 | -1.09 | -1.26 | | |
| **Putative aldehyde dehydrogenase** | A0A1E1X7S7 | | -0.70 | 0.60 | |
| **Putative lysine-ketoglutarate reductase/saccharopine dehydrogenase** | A0A1E1XF00 | | -0.60 | 0.73 | |
| **Tubulin alpha chain** | A0A1E1XQ11 | | -1.00 | 0.47 | |
| **Putative na+/h+ exchange regulatory cofactor nhe-rf1 (Fragment)** | A0A1E1XRK0 | | -0.91 | 0.61 | |
| **Insect cuticle domaincontaining protein** | B7PRW2 | | 0.60 | -0.66 | |
| **Cuticular protein, putative** | B7Q730 | | 0.76 | -0.83 | |
| **Cytochrome c oxidase subunit** | C9W1B7 | | -0.32 | 0.41 | |
| **ATP synthase E chain** | C9W1E1 | | -0.38 | 0.39 | |
| **Uncharacterized protein (Fragment)** | G3MHP9 | | -0.26 | 0.50 | |
| **Uncharacterized protein** | G3ML11 | | -0.59 | 0.53 | |
| **Uncharacterized protein** | G3MM71 | 0.20 | | -0.28 | -0.31 |
| **Uncharacterized protein** | G3MM94 | 0.45 | | 1.28 | |
| **Uncharacterized protein** | G3MMK9 | | -0.23 | | -0.37 |
| **Uncharacterized protein** | G3MNM0 | | 0.32 | -0.40 | |
| **Uncharacterized protein** | G3MPJ8 | | -0.69 | 0.29 | |

| Description | UniProt ID | Infection in unfed ticks | Infection in fed ticks | Feeding in uninfected ticks | Feeding in infected ticks |
|---|---|---|---|---|---|
| **Cuticular protein (Fragment)** | K7N8D7 | | 0.79 | -1.63 | |
| **Putative bitil peptide** | L7LTD3 | 0.64 | 1.06 | | |
| **Putative bitil peptide** | L7LTJ2 | 0.17 | | -0.64 | |
| **Putative heteroproteinous nuclear ribonucleoprotein at 87f** | L7LTQ8 | | -0.42 | 0.48 | |
| **Uncharacterized protein** | L7LUQ0 | | -0.65 | 0.28 | |
| **Putative m13 family peptidase** | L7LW70 | | -0.44 | 0.45 | |
| **Putative membrane atpase/protein kinase** | L7LWJ8 | 0.53 | | | -1.15 |
| **Putative molecular chaperones mortalin/pbp74/grp75 hsp70 superfamily** | L7LX08 | -0.48 | | 0.58 | |
| **Uncharacterized protein** | L7LXH0 | 0.46 | | 0.23 | |
| **Uncharacterized protein** | L7LZZ7 | | -1.30 | | -0.66 |
| **Putative na+/dicarboxylate na+/tricarboxylate and phosphate transporter** | L7M077 | | -0.99 | 0.72 | |
| **Putative ubiquitin fusion-degradation protein** | L7M0E6 | | -1.14 | 0.32 | |
| **Putative ubiquitin-conjugating enzyme e2** | L7M191 | | -0.63 | 0.37 | |
| **Putative scaffold attachment factor b** | L7M1L6 | | -0.51 | 0.21 | |
| **Putative ubiquitin-protein ligase** | L7M1X7 | 0.57 | -1.07 | 0.49 | -1.15 |
| **Peptidylprolyl isomerase (EC 5.2.1.8)** | L7M243 | | -0.59 | | -0.99 |
| **Putative mitochondrial import inner membrane translocase subunit tim8-like protein** | L7M332 | -0.53 | | -0.77 | |
| **Putative microtubule binding protein ytm1** | L7M334 | | -0.93 | 0.51 | |
| **Uncharacterized protein** | L7M3V8 | | -0.51 | 0.19 | |
| **Calcium load-activated calcium channel (CLAC channel)** | L7M4D2 | | -0.51 | 0.36 | |
| **Proteasome subunit alpha type (EC 3.4.25.1)** | L7M4S5 | | -0.41 | 0.32 | |
| **Uncharacterized protein** | L7M4W0 | | -0.47 | 0.27 | |
| **Peptidyl-prolyl cis-trans isomerase (PPIase) (EC 5.2.1.8)** | L7M551 | | -0.65 | 0.46 | |
| **Putative endocytosis/signaling protein ehd1** | L7M591 | | -0.61 | 0.59 | |
| **Putative vamp vesicle-associated membrane protein-associated protein a** | L7M5Q2 | | -0.26 | | -0.29 |
| **Putative bifunctional atp sulfurylase/adenosine 5'-phosphosulfate kinase** | L7M6S3 | | -0.63 | 0.40 | |
| **Putative kinesin light chain** | L7M6Y4 | | -0.34 | 0.32 | |

| Description | UniProt ID | Infection in unfed ticks | Infection in fed ticks | Feeding in uninfected ticks | Feeding in infected ticks |
|---|---|---|---|---|---|
| **Putative metalloexopeptidase** | L7M7B2 | | 0.50 | -1.10 | |
| **Putative biosynthetic process** | L7M7D6 | 0.14 | 0.22 | -0.10 | |
| **Putative skd/vacuolar sorting protein** | L7M7H3 | | -0.76 | 0.52 | |
| **Putative phenylalanyl-trna synthetase beta subunit** | L7M834 | | -0.65 | 0.40 | |
| **Phosphoglycerate kinase (EC 2.7.2.3)** | L7M8C7 | | -0.64 | 0.64 | |
| **Uncharacterized protein** | L7M8J0 | | 1.21 | -1.57 | |
| **Putative tick adams** | L7M8U8 | | 0.54 | -0.78 | |
| **Uncharacterized protein** | L7M8X0 | 0.35 | | 0.68 | |
| **Putative aquaporin major intrinsic protein family** | L7M963 | 0.13 | -0.56 | 0.78 | |
| **Putative erythrocyte membrane protein band 4.1-like 3** | L7M9D1 | | -0.26 | 0.28 | |
| **Putative metastriate ixostatin family member** | L7M9M0 | 0.70 | 0.69 | | |
| **Signal recognition particle subunit SRP72** | L7MA07 | | -0.37 | 0.19 | -0.39 |
| **Non-specific serine/threonine protein kinase (EC 2.7.11.1)** | L7MAI3 | | -0.46 | 0.46 | |
| **Putative elongation factor tu ef-tu** | L7MAK4 | | -0.57 | | -0.49 |
| **Uncharacterized protein (Fragment)** | L7MAU7 | 0.38 | -0.48 | 0.30 | -0.55 |
| **Putative transcription factor nfat subunit nf45** | L7MB29 | | -0.33 | 0.40 | |
| **Putative salivary lipase** | L7MBD3 | 1.03 | | -0.48 | -0.99 |
| **Putative cell division cycle 37 log (Fragment)** | L7MC52 | | -0.81 | 0.74 | |
| **Malate dehydrogenase (EC 1.1.1.37) (Fragment)** | L7MDB3 | 0.91 | | 0.79 | |
| **Putative pitrilysin metalloprotein (Fragment)** | L7MDT6 | | -0.49 | 0.51 | |
| **Glycogen [starch] synthase (EC 2.4.1.11) (Fragment)** | L7ME35 | | -1.01 | 0.51 | -0.76 |
| **Putative bilaris (Fragment)** | L7ME64 | | 0.33 | -0.37 | |
| **Putative atlastin-2 (Fragment)** | L7MFQ0 | 0.46 | -0.33 | 0.40 | |
| **Putative tick serine protease (Fragment)** | L7MG92 | | 0.65 | -0.78 | |
| **Putative glycine rich protein (Fragment)** | L7MGF8 | | -0.26 | | -0.35 |
| **Putative metalloprotease (Fragment)** | L7MGI7 | | -0.51 | 0.92 | |
| **Putative bitil peptide (Fragment)** | L7MHD8 | | 0.76 | -0.69 | |
| **Putative ca2+-dependent phospholipid-binding protein synaptotagmin required for synaptic vesicle (Fragment)** | L7MHQ5 | | -0.71 | | -0.72 |

| Description | UniProt ID | Infection in unfed ticks | Infection in fed ticks | Feeding in uninfected ticks | Feeding in infected ticks |
|---|---|---|---|---|---|
| **Putative tritil protein (Fragment)** | L7MI12 | | 0.93 | -1.29 | |
| **Putative dihydroorotase (Fragment)** | L7MIP5 | | -0.44 | 0.81 | |
| **Putative 8.9 kDa family member (Fragment)** | L7MJV2 | 0.58 | 0.84 | | |
| **Anion exchange protein (Fragment)** | L7MK92 | | -0.48 | 0.65 | |
| **Putative zn-ribbon-containing protein implicated in mitosis (Fragment)** | L7MM99 | -0.52 | | -0.32 | |
| **Glutathione peroxidase** | Q2XW18 | | -0.27 | 0.29 | |
| **Cathepsin L-like cysteine proteinase B** | Q7YW74 | | -0.52 | 0.82 | |
| **Putative microtubule-associated protein bicaudal-d (Fragment)** | V5GSM0 | | -0.65 | | -0.64 |
| **Putative golgi phosphoprotein 3 coat-protein gpp34 protein (Fragment)** | V5HUK3 | 0.60 | -0.54 | 0.56 | |
| **Putative ethylmalonyl-coa decarboxylase** | V5IH83 | | -0.65 | 0.57 | |
| **NADH-ubiquinone oxidoreductase chain 3 (EC 1.6.5.3)** | V9MMC1 | 0.54 | | 0.81 | |
| **Cytochrome c oxidase subunit 2** | V9MMC9 | 0.53 | | 0.33 | |

**Table S2. Primers used for double-stranded RNA synthesis.** RNAi assays were performed using specific primers containing T7 promoter sequences and annealing conditions.

| Gene (Abbreviation, UniProt ID) | Accession number | Primer Forward (5′-3′)* | Primer Reverse (5′-3′)* | Length (bp) | Annealing conditions (ºC) |
|---|---|---|---|---|---|
| *beta-2 microglobulin, β2M* **( P01887)**** | NM_009735 | CACCCCCACTGAGACTGATACA | AATTAGGCCTCTTTGCTTTACCA | 450 | 64 ºC |
| *Putative ubiquitin-protein ligase, UB2N* **(L7M1X7)** | GACK01007152 | CGGATTACCGAGGAGAATCA | CTTCTGCCTCGTTGACCTTC | 401 | 55 ºC |
| *Uncharacterized protein, PCCA* **(L7MAU7)** | GACK01003894 | AAGGCGCTTGATGCTTATGT | ACAAGGGGCAAGGAGAGATT | 407 | 64 ºC |

*All primers contained T7 promoter sequences (5′ – TAATACGACTCACTATAGGGAGA – 3′) at the 5′end. Primers were design using Primer3 platform (v 0.4.0, http://bioinfo.ut.ee/primer3-0.4.0/)[59] and its specificity predicted by PrimerBlast[60] (https://www.ncbi.nlm.nih.gov/tools/primer-blast/index.cgi). **An exogenous gene, mouse beta-2microglobulin (*β2M*) (GenBank: NM_009735) was used as control for the knockdown experiments.

**Table S3. Primers used for gene knockdown assessment.** The sequences of the primers designed for two targets and three putative reference genes and respective conditions.

| Gene (Abbreviation, UniProt ID) | Accession number | Primer Forward (5′-3′)* | Primer Reverse (5′-3′)* | Length (bp) | Annealing conditions (ºC, µM) |
|---|---|---|---|---|---|
| ***Putative ubiquitin-protein ligase, UB2N* (L7M1X7)** | GACK01007152 | CCACCCCAACATAGACAAGC | CTCGCTGTTCTGATGGCTTC | 195 | 59.2, 0.5 |
| ***Uncharacterized protein, PCCA* (L7MAU7)** | GACK01003894 | CCAGGATTTGATGGCGTAGT | GTCTCCGAAGCTTGACTTGG | 189 | 54.5, 0.8 |
| ***16S rRNA***** | 61 | GACAAGAAGACCCTA | ATCCAACATCGAGGT | 212 | 56, 0.5 |
| ***elongation factor***** | 62 | CGTCTACAAGATTGGTGGCATT | CTCAGTGGTCAGGTTGGCAG | 109 | 62.5, 0.5 |
| ***β-tubulin***** | 62 | AACATGGTGCCCTTCCCACG | GCAGCCATCATGTTCTTTGC | 140 | 60.5, 0.5 |

*Primers were design using Primer3 platform (v 0.4.0, http://bioinfo.ut.ee/primer3-0.4.0/)[59] and its specificity predicted by PrimerBlast[60] (https://www.ncbi.nlm.nih.gov/tools/primer-blast/index.cgi). **Used as reference gene for gene expression analysis.

**Table S4. Primers used for detection and quantification of *Babesia ovis*.** The sequences of the primers designed for *Babesia* detection in the host (*Ovis aries*) and vector (*Rhipicephalus bursa*) and respective conditions.

| Gene (Species) | Accession number | Primer Forward (5′-3′)* | Primer Reverse (5′-3′)* | Length (bp) | Annealing conditions (ºC, µM) |
|---|---|---|---|---|---|
| ***BoSPD* (*Babesia ovis*)**** | 10 | GCTGGGCGTTAATCAGAGAG | GTGTTCGTCTTCGTCCCAGT | 151 | 54.5, 0.8 |
| ***Ov18s* (*Ovis aries*)**** | 65 | GCAATTATTCCCCATGAACG | CAAAGGGCAGGGACTTAATC | 77 | 56, 0.3 |
| ***16S rRNA* (*Rhipicephalus bursa*)**** | 61 | GACAAGAAGACCCTA | ATCCAACATCGAGGT | 212 | 59.6, 0.5 |

*Primers were design using Primer3 platform (v 0.4.0, http://bioinfo.ut.ee/primer3-0.4.0/)[59] and its specificity predicted by PrimerBlast[60] (https://www.ncbi.nlm.nih.gov/tools/primer-blast/index.cgi). **gBlocks® Gene Fragements (Integrated DNA Technologies, Leuven, Belgium) was synthetized for each target sequence
